# Comparison of Survival Outcomes between Minimally Invasive Surgery and Open Radical Hysterectomy in Early-Stage Cervical Cancer

**DOI:** 10.3390/cancers14092117

**Published:** 2022-04-24

**Authors:** I.-Ning Chen, I.-Te Wang, Hsueh-Yu Mu, J.-Timothy Qiu, Wei-Min Liu, Ching-Wen Chang, Yen-Hsieh Chiu

**Affiliations:** 1 Department of Obstetrics and Gynecology, Taipei Medical University Hospital, Taipei 110301, Taiwan; ningchendr@gmail.com (I.-N.C.); b8301120@yahoo.com.tw (I.-T.W.); hsuehyu0220@gmail.com (H.-Y.M.); jtqiu@hotmail.com (J.-T.Q.); weiminliu50@gmail.com (W.-M.L.); ching967@yahoo.com.tw (C.-W.C.); 2 Department of Obstetrics and Gynecology, College of Medicine, Taipei Medical University, Taipei 110301, Taiwan; 3 International PhD Program of Cell Therapy and Regenerative Medicine, Taipei Medical University, Taipei 110301, Taiwan

**Keywords:** cervical cancer, hysterectomy, laparotomy radical hysterectomy, minimally invasive surgery, laparoscopic surgery, robotic-assisted surgery

## Abstract

**Simple Summary:**

Radical hysterectomy is one of the most crucial treatments for early-stage cervical cancer. However, which surgical approach is better, either minimally invasive surgery (MIS) or laparotomy, is still debatable. This study showed comparative survival outcomes between MIS and laparotomy radical hysterectomy and provided some viewpoints on selected cases. Subgroup analyses showed favorable survival outcomes in patients who received robotic-assisted surgery and had pathologic tumor size ≤ 2cm. With advancements in surgical technologies and comprehensions of pelvic anatomy regarding to the parametrium and the paracolpium, MIS has gradually become an alternative for early-stage cervical cancer. Moreover, for patients with tumor size > 2cm, laparotomy radical hysterectomy showed good radicality in the resection of parametrium. We concluded that precise clinical evaluations and tailored surgical approaches for patients with early-stage cervical cancer were of great significance.

**Abstract:**

Objectives: To compare the survival outcomes between minimally invasive surgery (MIS) and laparotomy radical hysterectomy in patients with early-stage cervical cancer. Methods: We conducted a retrospective study involving women who received a radical hysterectomy for cervical cancer, stage IA1 with lymphovascular invasion, IA2, IB1, IB2, or IIA from 2008 to 2016. Clinicopathologic and perioperative outcomes were compared using appropriate statistical methodologies. Results: Oncologic survival outcomes were analyzed using the Kaplan–Meier method. Among the 105 cases identified, 58 (55.2%) and 47 (44.8%) women underwent MIS and open radical hysterectomy, respectively. Over a median follow-up period of 62 months, women who underwent MIS and open radical hysterectomy had a 5-year overall survival rate of 87.9% and 89.4% (*p* = 0.845) and a 5-year disease-free survival rate of 82.5% and 86.7% (*p* = 0.624), respectively. Conclusions: For early-stage cervical cancer, patients who underwent MIS radical hysterectomy had survival outcomes that were comparable to those who underwent open surgery at our institute.

## 1. Introduction

According to the National Comprehensive Cancer Network (NCCN) guidelines [1], radical hysterectomy is one of the standard treatments for early-stage cervical cancer. However, which surgical approach is better, either minimally invasive surgery (MIS) or laparotomy, remains uncertain.

After the first case of laparoscopic radical hysterectomy and para-aortic and pelvic lymphadenectomy was reported [2], other surgical options for cervical cancer have become available. In Sweden, the robotic surgical technique has gradually replaced open surgery for cervical cancer at most centers [3]. Some systemic reviews reported that minimally invasive radical hysterectomy was associated with a shorter hospital stay, lesser blood loss, faster recovery, and fewer complications compared with open surgery [4,5,6]. Moreover, multiple retrospective studies showed comparable survival outcomes between both approaches [7,8,9,10].

Nonetheless, two recent publications in 2018 reported that MIS had worse survival outcomes compared with laparotomy surgery, raising concerns regarding the safety of MIS for the treatment of early-stage cervical cancer. One large retrospective national cancer database study in the US noted that MIS promoted shorter survival compared with laparotomy [hazard ratio (HR) 1.65, *p* < 0.002] [11]. Similarly, another randomized controlled trial, the Laparoscopic Approach to Cervical Cancer (LACC) trial [12,13], reported that the MIS arm exhibited a higher 4-year mortality rate compared with the open arm (9.1% vs. 5.3%, HR, 1.65, *p* = 0.002). The aforementioned data resulted in a change in the treatment guidelines for patients with early-stage cervical cancer by the NCCN [14] and the European Society of Gynecological Oncology [15].

In Taiwan, the age-adjusted incidence rate of cervical cancer is approximately 7.9 of 100,000. Approximately 1433 new cases of cervical cancer were diagnosed in 2018, with 653 women dying of this disease according to the Health Promotion Administration, Ministry of Health and Welfare, R.O.C. (Taiwan). Moreover, the 5-year survival rate of stage 1 cervical cancer from 2012 to 2016 was 84.6%. The surgical approach for early-stage cervical cancer is surgeon-dependent to a large extent.

This study aimed to compare the survival outcomes, including 5-year overall survival (OS), 5-year disease-free survival (DFS), pattern of recurrence, and perioperative outcomes, between patients who underwent MIS and open radical hysterectomy for early-stage cervical cancer from 2008 to 2016.

## 2. Materials and Methods

This retrospective study was conducted at Taipei Medical University Hospital, Taiwan, ROC. To ensure the quality of data on follow-up, a chart review using defined criteria for relevant clinical parameters was performed on the complete cohort of women with cervical cancer from January 2008 to December 2016. We included patients with pathologically confirmed early-stage cervical carcinoma from stage IA1 with lymphovascular invasion, IA2, IB1, IB2, and IIA who underwent radical hysterectomy. All patients received pelvic lymphadenectomy rather than sentinel lymphadenectomy. Patients who received any preoperative chemotherapy, radiation therapy, or chemoradiation therapy were excluded. We also excluded cervical cancer cases with a histologic subtype of neuroendocrine cells. All medical reviews were performed to obtain clinicopathological, operative, and survival data, including age, body mass index, clinical stage of disease [16], tumor histologic subtypes, pathological tumor size, positive lymph nodes, positive surgical margins, positive lymphovascular invasion, resected length of vaginal cuff, resection area of the left and right parametrium, residual tumor in the hysterectomy specimen, surgical approach, types of radical hysterectomies [17], adjuvant therapy, time from surgery to death or last follow-up, time from surgery to recurrence, and sites of recurrence. With regard to the types of hysterectomies, type B, type C1 [18], and type C2 radical hysterectomies were performed according to both the surgeon’s preference and NCCN guidelines.

All continuous variables were analyzed using the independent Student’s *t*-test, whereas the categorical variables were analyzed using Pearson’s chi-squared test or Fisher’s exact test depending on the category size. Survival curve analyses were performed using the Kaplan–Meier method, and comparisons were made using the log-rank test while calculating the hazard ratio (HR), the accompanying 95% confidence interval (CI), and the *p* value. OS was defined as the time from surgery to the time of death from any cause. Patients who survived were censored at the time of the last follow-up. DFS was defined as the time from surgery to the time of the first recurrence. Patients without recurrence were censored at the time of the last follow-up or upon non-disease-related death. Cox regression analysis was performed to create univariate and multivariate models for DFS and OS to account for potential confounders. A *p* value of <0.05 was considered statistically significant. All statistical analyses were performed using IBM SPSS Statistics version 18.0.7.

## 3. Results

During the period from 2008 to 2016, a total of 105 women underwent a radical hysterectomy for stage IA1 with lymphovascular invasion, IA2, IB1, IB2, or IIA1 cervical carcinoma at our hospital. Among these women, 58 (55.2%) received MIS, whereas 47 (44.8%) received laparotomy surgery. Among the MIS group, 50% of the patients (*n* = 29) underwent robotic-assisted surgery, whereas others underwent laparoscopic-assisted surgery (*n* = 29).

The baseline characteristics are summarized in Table 1. No significant difference in median age [48.5 years (range 31–89 years) and 52.0 years (range 37–74 years), *p* = 0.634], median BMI [23.1 kg/m^2^ (range 17.2–30.2 kg/m^2^) and 22.7 kg/m^2^ (range 16.2–34.5 kg/m^2^), *p* = 0.887] and median vaginal cuff length [1.62 cm (range 0.2–7.0 cm and 1.76 cm (range 0.5–6.0 cm), *p* = 0.55] were observed between the MIS and open groups. Moreover, no difference in clinical stage of disease, subtype of histology, positive nodes, positive margins, presence of lymphovascular invasion, adjuvant therapy, or residual tumors in the specimen were observed between both groups. Most patients in both groups had clinical stage IB1 (69.1% and 63.0% in the MIS and open groups, respectively, *p* = 0.188) and squamous carcinoma in the histologic subtype (56.9% and 70.2% in the MIS and open groups, respectively, *p* = 0.658). Notably, patients with clinical stage IIA1 took the second and third place (14.5% vs. 15.2%) in the MIS group and open group, respectively. The MIS group had a significantly smaller median pathologic tumor size compared to the open group (2.30 vs. 3.15 cm, *p* = 0.01). The MIS group had a significantly smaller parametrium resection area (5.54 vs. 8.84 cm^2^ in the right parametrium area, *p* = 0.002; 5.24 vs. 7.58 cm^2^ in the left parametrium area, *p* = 0.007) when compared with the open group.

**Figure 1:**
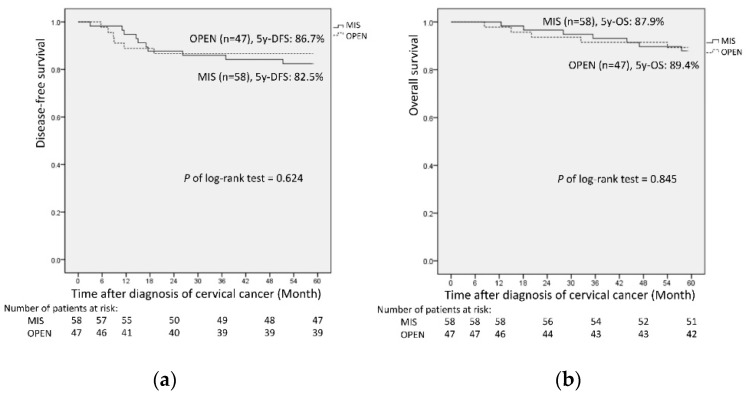
Oncologic outcomes of minimally invasive surgery (MIS) versus open radical hysterectomy: (**a**) 5-year disease-free survival; (**b**) 5-year overall survival.

The median follow-up time in this study was 62 months and ranged from 8.3 to 161 months (median follow-up time, 60.5 and 74.0 months in the MIS and open groups, respectively, *p* = 0.127). All patients in this study were followed up for at least 60 months, with the exception of the patients who died during the follow-up period. No significant difference was observed in the survival outcomes between the groups. Throughout the study period, 10 recurrences and 7 deaths in the MIS group and 6 recurrences and 5 deaths in the open group were observed. The open group had a lower recurrence rate (12.8% vs. 17.2%, *p* = 0.562) and death rate (10.6% vs. 12.1%, *p* = 0.819) compared to the MIS group (Table 2). The sites of recurrence are detailed in Table 3. Notably, no significant difference was noted in the survival rates between the groups. Both the 5-year OS rate and 5-year DFS rate were higher in the open group than in the MIS group (5 y-OS: 89.4% vs. 87.9%, *p* = 0.845 and 5 y-DFS: 86.7% vs. 82.5%, *p* = 0.624; Figure 1). The median survival time was not reached.

**Figure 2:**
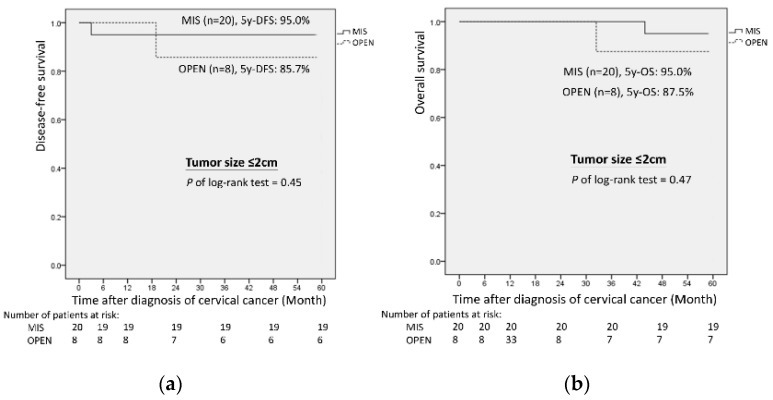
Oncologic outcomes of MIS versus open radical hysterectomy with pathologic tumor size ≤ 2 cm: (**a**) 5-year disease-free survival; (**b**) 5-year overall survival.

**Figure 3:**
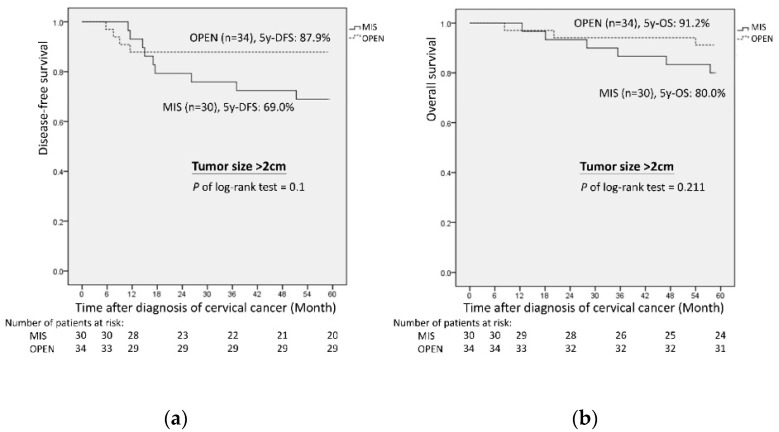
Oncologic outcomes of MIS versus open radical hysterectomy with pathologic tumor size > 2 cm: (**a**) 5-year disease-free survival; (**b**) 5-year overall survival.

Among patients with a pathologic tumor size ≤ 2 cm, the MIS group showed a higher 5-year OS and 5-year DFS when compared with the open group (5-year OS: 95.0% vs. 87.5%, *p* = 0.47 and 5-year DFS: 95.0% vs. 85.7%, *p* = 0.45; Figure 2), although the differences were not statistically significant. However, among patients with a pathologic tumor size > 2 cm, the open group had a better 5-year OS and 5-year DFS when compared with the MIS group (OS: 91.2% vs. 80.0%, *p* = 0.21 and DFS: 87.9% vs. 69.0%, *p* = 0.10; Figure 3).

After adjusting for all possible confounding factors, including surgical approach (open/MIS), age, clinical stage of disease, histologic subtype (adenocarcinoma and adenosquamous carcinoma/squamous cell carcinoma), pathologic tumor size (>2 cm/≤2 cm), positive margin (yes/no), LVI (yes/no), positive node (yes/no), adjuvant therapy (yes/no), resected length of vaginal cuff, resected area of the right parametrium, and resected area of the left parametrium using a univariate and multivariate Cox regression analysis, the adjusted HR (open/MIS) for recurrence and mortality were 1.27 (95% CI: 0.37–4.41) and 1.18 (95% CI: 0.18–8.48), respectively, which were both not statistically significant (*p* = 0.71 and *p* = 0.87; Table 4).

A univariate regression analysis with OS as the end-point showed that the histologic subtype of the adenocarcinoma component, positive lymphovascular invasion, and positive margin were significantly associated with higher HR, but the significance of positive lymphovascular invasion and positive margin disappeared during multivariate analysis, as shown in Table 4. Only the histologic subtype of the adenocarcinoma component (HR 27.25, 95% CI: 4.4–167.4, *p* < 0.05) was found to be a significant independent risk factor. Moreover, with DFS as the end-point, a multivariate regression showed that a higher HR in the histologic subtype of the adenocarcinoma component (HR 7.6, 95%CI: 2.3–25.2, *p* = 0.001), and positive lymphovascular invasion (HR 45.5, 95%CI: 3.3–635.4, *p* = 0.005) were significant independent risk factors.

Subgroup analyses of the survival between laparoscopic between robotic-assisted surgery and robotic-assisted surgery between open surgery were done. In particular, among patients with a pathologic tumor size ≤ 2 cm, a higher 5-year OS and 5-year DFS in the robotic group compared with laparoscopic surgery were noted (5-year OS: 100% vs. 88.9%, *p* = 0.699 and 5-year DFS: 100% vs. 88.9%, *p* = 0.52), although the differences were not statistically significant. However, among patients with a pathologic tumor size > 2 cm, a higher 5-year OS rate (82.4% vs. 76.9%, *p* = 0.728) and lower 5-year DFS rate (64.7% vs. 75.0%, *p* = 0.495) were noted in the robotic group compared to laparoscopic surgery.

Of note, 11 patients with a tumor size ≤2 cm in the robotic group all survived without experiencing recurrence. Furthermore, when the robotic group was compared with the open group, higher survival rates were also noted (5-year OS: 100% vs. 87.5%, *p* = 0.241 and 5-year DFS: 100% vs. 85.7%, *p* = 0.21). However, among patients with a pathologic tumor size > 2 cm, the open group had better 5-year OS and DFS rates when compared with the robotic group (OS: 91.2% vs. 82.4%, *p* = 0.375 and DFS: 87.9% vs. 64.7%, *p* = 0.77).

## 4. Discussion

This study showed comparable survival outcomes between MIS and open radical hysterectomy in terms of both 5-year DFS and OS rates, which contradict the results of the two studies published in the New England Journal of Medicine in 2018 [11,12]. Nonetheless, our findings were consistent with the results of previous studies [3,4,5,6,7,8,9,10,19].

Although no significant survival differences were observed between the two groups, our data showed that, among patients with a pathologic tumor size ≤ 2 cm, the MIS group had longer OS (95.0% vs. 85.7%) and DFS (95% vs. 87.5%) than the open group (Figure 2). Furthermore, the survival outcomes among patients with a smaller tumor size in the MIS group were comparable to those in previously published studies [20,21,22,23]. Our findings were also consistent with the 2018 International Federation of Gynecology and Obstetrics (FIGO) staging system of cervical cancer [24,25], which introduced a new primary tumor size cutoff value of 2 cm (i.e., stage IB1 vs. IB2) in order to estimate the prognosis and evaluate the possibility of fertility sparing surgery. In other words, in selected cases, especially for patients with tumor sizes ≤ 2 cm who showed comparable survival outcomes, MIS may be considered a feasible alternative to open surgery for early-stage cervical cancer.

The baseline characteristics revealed that the open group had significantly larger tumor sizes than the MIS group. As such, a selection bias between the two groups may have been present, considering that larger tumor sizes are indicative of more complicated surgical procedures, longer operative time, increased metastatic risk, or require advanced surgical technique during MIS. Therefore, our surgeons tended to perform laparotomy radical hysterectomy in patients with larger tumor sizes. We noted that when the pathologic tumor size was >2 cm, a better OS (87.9% vs. 69.0%) and DFS (91.2% vs. 80.0%) were noted in the open group (Figure 3). This suggests that laparotomy radical hysterectomy might be a better surgical option for patients with tumor sizes > 2 cm. This conclusion is also consistent with the outcomes of the SUCCOR study [26].

An adequate resection margin of the parametrium is required for a better prognosis. In fact, Lee et al. believed that adequate radicality and resection of early-stage cervical cancer accounted for excellent survival outcomes [27]. They reported excellent survival data with both 100% 5-year OS and 100% 5-year DFS among 53 patients that received laparoscopic radical hysterectomy. Our research also showed significantly larger parametrium resection areas in the open group for both the right (open: 8.84 cm^2^ vs. MIS: 5.54 cm^2^, *p* = 0.002) and left regions (open: 7.58 cm^2^ vs. MIS: 5.24 cm^2^, *p* = 0.007). After referring to the results of another similar study [20], we believe that the resection of the parametrial area could preferably not be less than 6.3 cm^2^, particularly in patients with tumor sizes > 2 cm.

With respect to the resected length of the vaginal cuff, the NCCN guideline suggests a vaginal length of 1.0 to 2.0 cm for type B radical hysterectomy and that of at least 2 cm dorsal from the cervix for type C1 radical hysterectomy [1]. A retrospective study [28] deemed that the resection length of a vaginal cuff > 2.0 cm in radical hysterectomy had a more favorable long-term outcome than that of the vaginal cuff length when it is ≤ 2.0 cm, whereas another similar study [29] stated otherwise. Our data displayed that 22 patients (23.4%) with a resected length of a vaginal cuff > 2.0 cm had a higher 5-year overall survival rate when compared with those with a resected length of a vaginal cuff ≤ 2 cm (95.5% vs. 86.1%, *p* = 0.23; HR, 0.31; 95%CI, 0.04–2.39, *p* = 0.24). Although it showed no statistical difference, there appeared to be a trend for better survival outcomes in patients with a resected length of a vaginal cuff > 2 cm. There is still room for improvement in the surgical techniques used for the resection length of vaginal cuff.

Our investigation showed that three patients who had a pathologic tumor size of ≤2 cm experienced tumor recurrence and/or death because of clinically under-staged FIGO status and/or unsatisfied surgery. One woman in the MIS group who had a recurrent tumor located in the para-aortic lymph node after 3 months, which was detected under computed tomography, died due to lung and thoracic cage metastasis 43.9 months later. Given the lack of pre-operative imaging studies on lymph node status, the patient was assumed to have had a smaller cervical tumor with parametrial involvement and lymph node metastasis before surgery. Rongxu Du et al. disclosed that a positive para-aortic lymph node may have contributed to a significantly higher HR for DFS and OS (4.1 and 5.1, respectively) [30]. Another woman who received a laparotomy radical hysterectomy exhibited tumor recurrence at the vaginal cuff 19 months later. We noticed that the vaginal cuff length in the specimen was 1.4 cm. Reports have shown that resection was significantly associated with local recurrence, DFS, and OS among those with a shorter vaginal cuff length (i.e., <2 cm) [27,28]. The other patient died 32.4 months after open surgery and had a pathologic tumor size of 1.2 cm, but had a FIGO stage of IIIC, owing to positive pelvic lymph nodes. Generally, early cervical cancer has a 12–29% positivity rate for lymphatic metastasis [20,26,30], indicating the importance of detailed pre-operative imaging and precise clinical staging.

A patient with adenosquamous cell carcinoma had a poor survival outcome (DFS and OS of 11 and 12.5 months, respectively). One case with basal cell carcinoma of cervical cancer was excluded. This histologic subtype is very rare in cervical cancer, with only a few case reports and case series having been reported [31]. Given its rarity and relatively favorable outcomes compared with squamous cell carcinoma, adenosquamous carcinoma or adenocarcinoma was excluded in the current study.

Some limitations of the current study are worth noting. This was a retrospective study with a relatively small number of patients. A few baseline differences were present between both groups, which we attempted to adjust using a multivariate model and subgroup analyses.

## 5. Conclusions

Our study showed comparable survival outcomes for both OS and DFS between patients undergoing MIS and open radical hysterectomy. We suggest a precise clinical evaluation prior to surgery, including imaging studies for pre-operative lymph node status. We also recommend MIS radical hysterectomy for selected cases, particularly in patients with tumor sizes ≤ 2 cm. Nonetheless, for patients with tumor sizes > 2 cm, open surgery with good radicality and an adequate resection of the parametrium still remain critical.

## Figures and Tables

**Table 1:** Baseline clinicopathologic characteristics of minimally invasive surgery (MIS) versus open radical hysterectomy.

Variable	MIS *n* (%)	Open *n* (%)	*p*-Value
Number	58 (55.2)	47 (44.8)	
Approaches			
Laparoscopic	29 (50)		
Robotic	29 (50)		
Age, years Median (range)	48.5 (31–89)	52 (37–74)	0.63
BMI, kg/m^2^ Median (range)	23.1 (17.2–30.2)	22.7 (16.2–34.5)	0.89
Clinical Stage			
IAI with LVI	0 (0)	0 (0)	0.19
IA2	6 (10.9)	2 (4.3)	
IB1	38 (69.1)	29 (63.0)	
IB2	3 (5.5)	8 (17.4)	
IIA1	8 (14.5)	7 (15.2)	
Histology			
SCC	33 (56.9)	33 (70.2)	0.66
Adenocarcinoma	18 (31.0)	12 (25.5)	
Adenosquamous	1 (1.7)	1 (2.1)	
Unknown	6 (10.3)	1 (2.1)	
Pathologic tumor size, cm Median (range)	2.3 (0.4–5.8)	3.15 (0.5–10)	0.01
Pathologic tumor size			0.03
≤2 cm	20 (40)	8 (19)	
>2 cm	30 (60)	34 (81)	
Positive margin	7 (12.1)	7 (15)	0.67
LVI	27 (46.6)	27 (57.4)	0.29
Positive node	10 (17.2)	12 (25.5)	0.30
Resected length of the vaignal cuff, cm			0.55
Median(range)	1.62 (0.2–7.0)	1.76 (0.5–6.0)	
Rt parametrium area, cm^2^ Median (range)	5.5 (0.1–16.5)	8.8 (1.8–32.0)	0.002
Lt parametrium area, cm^2^ Median (range)	5.2 (0–17.0)	7.6 (0–20.0)	0.007
Adjuvant therapy	8 (13.8)	6 (12.8)	0.88
No residual in hysterectomy	4 (9.8)	1 (6.3)	0.29

MIS, minimally invasive surgery; BMI, body mass index; FIGO, International Federation of Gynecology and Obstetrics: LVI, lymphovascular invasion; SCC, squamous cell carcinoma; Rt, right; Lt, left.

**Table 2:** Oncologic survival outcomes of MIS versus open radical hysterectomy.

Variable	MIS *n* (%)	Open *n* (%)	*p*-Value
Number	58 (55.2)	47 (44.8)	
Follow-up, months	60.5 (12.5–161)	74 (8.3–162)	0.13
Median			
Recurrence	10 (17.2)	6 (12.8)	0.56
Death	7 (12.1)	5 (10.6)	0.82
5-yr DFS rate	82.5%	86.7%	0.624
5-yr OS rate	87.9%	89.4%	0.845
Tumor ≤ 2 cm			
Number	20	8	
5-yr DFS rate	95.0%	85.7%	0.45
5-yr OS rate	95.0%	87.5%	0.47
Tumor > 2 cm			
Number	30	34	
5-yr DFS rate	69.0%	87.9%	0.10
5-yr OS rate	80.0%	91.2%	0.21

MIS, minimally invasive surgery; DFS, disease-free survival; OS, overall survival. DFS and OS presented as percent and standard error.

**Table 3:** Sites of recurrence.

Variables	MIS *n* (%)	Open *n* (%)	*p*-Value
Number	10 (17.2)	6 (12.8)	
Vaginal cuff alone	0 (0)	1 (16.7)	0.2
RP LNs	3 (30.0)	1 (16.7)	
Pelvic organ, non-cuff	0 (0)	2 (33.3)	
Abdomen	4 (40.0)	1 (16.7)	
Distant (liver, lung, bone)	1 (10.0)	1 (16.7)	
Multiple site	2 (20.0)	0 (0)	

MIS, minimally invasive surgery; RP LN, retroperitoneal lymph nodes.

**Table 4:** Univariate and multivariate regression analysis with overall survival and disease-free survival as the end-point.

Variable	DFS/Univariate Regression Analysis		DFS/Multivariate Regression Analysis		OS/Univariate Regression Analysis		OS/Multivariate Regression Analysis	
	HR (95% CI)	*p*-Value	HR (95% CI)	*p*-Value	HR (95% CI)	*p*-Value	HR (95% CI)	*p*-Value
Approach (Open/MIS)	0.98 (0.28–2.14)	0.63	1.27 (0.37–4.41)	0.71	0.89 (0.28–2.81)	0.85	1.18 (0.17–8.48)	0.87
Age	0.78 (0.94–1.02)	0.34	0.93 (0.85–1.01)	0.09	0.98 (0.93–1.03)	0.43	0.94 (0.85–1.04)	0.22
Clinical Stage								
IA2/IB1	0.00 (0–.)	0.98	0.00 (0–.)	0.99	0.00 (0–.)	0.99	0.00 (0–.)	1.00
IB2/IB1	0.47 (0.06–3.56)	0.46	5.84 (0.36–93.9)	0.21	0.56 (0.07–4.35)	0.58	12.52 (0.47–330.81)	0.13
IIA1/IB1	0.63 (0.14–2.81)	0.63	0.87 (0.14–5.22)	0.88	0.00 (0–.)	0.98	0.00 (0–.)	0.98
Histology Adeno ^a^/SCC	2.39 (0.90–6.37)	0.08	7.60 (2.3–25.19)	0.001	4.6 (1.39–15.29)	0.013	27.25 (4.44–167.36)	0.00
Tumor size (>2 cm/≤2 cm)	3.00 (0.68–13.31)	0.15	1.33 (0.75–2.34)	0.33	2.06 (0.44–9.52)	0.36	0.57 (0.22–1.43)	0.23
Positive margin (yes/no)	2.73 (0.88–8.48)	0.08	45.47 (3.25–635.43)	0.005	5.39 (1.71–17.05)	0.004	8.80 (0.66–116.7)	0.10
LVI (yes/no)	7.11 (1.62–31.29)	0.009	0.33 (0.06–1.73)	0.19	4.68 (1.39–15.29)	0.046	7.51 (0.71–79.04)	0.93
Postive node (yes/no)	1.49 (0.28–4.62)	0.49	3.30 (0.48–22.6)	0.22	2.94 (0.93–9.26)	0.066	6.64 (0.61–72.32)	0.12
Adjuvant therapy (yes/no)	2.83 (0.74–7.08)	0.15	0.36 (0.05–2.41)	0.29	2.34 (0.63–8.66)	0.20	3.02 (0.35–26.13)	0.32
Resected length of vaginal cuff	0.83 (0.5–1.41)	0.5	0.75 (0.37–1.5)	0.42	0.60 (0.28–1.29)	0.19	0.45 (0.13–1.58)	0.22
Right parametrium area Left parametrium area	0.91 (0.8–1.03)	0.13	0.89 (0.74–1.07)	0.2	0.94 (0.83–1.09)	0.44	1.01 (0.83–1.24)	0.92
	0.99 (0.88–1.11)	0.82	0.94 (0.77–1.15)	0.55	0.98 (0.86–1.13)	0.8	0.95 (0.73–1.23)	0.69

MIS, minimally invasive surgery; FIGO, International Federation of Gynecology and Obstetrics: LVI, lymphovascular invasion; SCC, squamous cell carcinoma; HR, hazard ratio; CI, confidence interval; LVI, lymphovascular invasion; ^a^ Adenocarcinoma and adenosquamous carcinoma.

## Data Availability

The data contained in the article cannot be shared due to data protection regulations. According to the ethics committee, only evaluation of anonymized data is allowed for this study.
